# Advances in Betalain Biosynthesis and Metabolic Engineering for Sustainable Natural Pigment Production

**DOI:** 10.3390/biotech15030056

**Published:** 2026-07-19

**Authors:** Saravanan Monisha, Marimuthu Kanchana, Aiyar Balasubramanian, Rajendran K. Selvakesavan

**Affiliations:** 1 Department of Botany, PSGR Krishnammal College for Women, Coimbatore 641004, India; 23phdby001@psgrkcw.ac.in; 2 Plant Biotechnology and Cytogenetics Division, Institute of Forest Genetics and Tree Breeding, Coimbatore 641002, India; balabio@gmail.com; 3 Department of Biotechnology, PSGR Krishnammal College for Women, Coimbatore 641004, India

**Keywords:** natural pigments, betalain biosynthesis, bio-factory, elicitation strategies, metabolic engineering, bioproduction

## Abstract

Betalains are water-soluble pigments containing nitrogen, and they exist naturally in the plants of the order Caryophyllales. They have gained increasing attention in recent years because of their intense colours, antioxidant activity, and safety, thus making them suitable replacements for artificial dyes. The increasing interest in natural pigments has led to intensified research on betalain biosynthesis and optimization of pigment production. Nonetheless, their application in industry faces limitations, such as their low natural occurrence, sensitivity to environmental conditions, and instability during manufacturing and storage. Unlike previous reviews that primarily focused on betalain chemistry, biosynthesis pathways, or biological activity, the present review highlights recent developments in the engineering of the biosynthesis pathways, synthetic biology, elicitation approaches, omics-based pathway identification, and nanobiotechnology for betalain pigments. Special attention is paid to the comparison of plant, plant cell, yeast, and bacterial production systems, as well as recent advancements towards industrial production of betalain pigments and bottlenecks in the commercialization of sustainable betalain bio-factories.

## 1. Introduction

Among all the attributes that determine consumer acceptance of foods, pharmaceuticals, cosmetics, and nutraceuticals, colour is probably the foremost. Apart from adding to the esthetic appeal of any food or cosmetic, colours also play an extremely vital role in determining perceptions about freshness, nutritive value, safety, and quality of a product by consumers. The use of synthetic colourants in industrial operations for years now has mainly been due to their low cost, high colouring power, wider availability of colours, and better stability of synthetic colourants as compared to other natural colourants used in industrial applications [1,2]. Currently, synthetic pigments have dominated the global markets due to their prevalence, as they account for an approximate market share of 76%, whereas natural pigments held a market share of only 24% in 2022 [3]. Global sales of synthetic dyes have reached USD 7.1 billion as of 2024, which will rise to USD 9.1 billion by 2029, with a compound annual growth rate (CAGR) of 5.0% [4]. However, growing consumer awareness about the various harmful impacts of several artificial colours on both health and the environment has led to greater consumer inclination toward using natural colourants. This has led to the development of plant-based pigments, which have become of great significance in food sciences, biotechnology, and agricultural and industrial applications. Natural pigments such as carotenoids, anthocyanins, chlorophylls, and betalains provide colour while offering antioxidant activity, biodegradability, biocompatibility, and low toxicity [5]. The rapid expansion of the market for natural colourants is attributed to the changes in policy regulations regarding food additives and growing interest in sustainable products.

**Figure 1:**
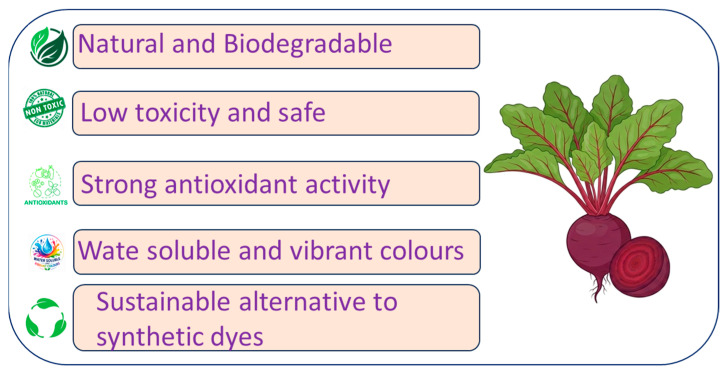
Key advantages of betalains, including antioxidant activity, water solubility, safety, and industrial applicability.

From the various classes of natural pigments, the betalains have attracted significant attention in terms of research and industrial applications due to their colourful nature, solubility in water, and adaptability to current sustainable production technologies. The betalains represent a group of nitrogenous pigments derived from L-tyrosine and are exclusively found in plants that belong to the Caryophyllales order [6]. These pigments are characterized by bright red-violet and orange-yellow hues, thus making them desirable colourants in the food and cosmetics industries. More importantly, betalains possess high antioxidant activity and a favourable safety profile, making them promising natural alternatives to synthetic colourants in the food and other industrial sectors (Figure 1). Representative synthetic food colourants that could potentially be replaced by betalains and their reported toxicological concerns are summarized in Table 1. Nevertheless, there are limitations concerning the application of these pigments, including low yields, limited natural sources, stability issues, and environmental sensitivities. Some of the notable issues that have been highlighted about betalain commercialization include limited supply sources of these pigments. While red beet (*Beta vulgaris*) continues to be the most common source for industrial use, reliance on few crops for betalains creates sustainability challenges, vulnerability to climatic variations, seasonality, and poor pigment quality. In addition, the production of betalains in plants is highly sensitive to environmental factors like temperature, light, salinity, nutritional levels, and biotic/abiotic stresses. Such variability often leads to fluctuations in pigment yield and quality, thereby affecting industrial scalability and economic feasibility [7]. Another major limitation of betalains is their physicochemical instability. Betalains are extremely sensitive to factors such as heat, oxygen, pH, light, and enzymatic reactions [8]. In the process of industrial production and storage, betalains can oxidize, decompose, or hydrolyze. As a result, the pigmentation properties of these substances decrease, which greatly limits their use in thermally treated food products. Thus, improvement of betalain stability and synthesis efficiency is currently one of the key issues of pigment biotechnology.

Recent advances in plant biotechnology, metabolic engineering, synthetic biology, and nanotechnology have created new opportunities to overcome the limitations associated with betalain production. The identification of key biosynthetic enzymes, including cytochrome P450, DOPA dioxygenase (DODA), and glucosyltransferases, as well as their regulatory transcription factors, has significantly improved our understanding of the molecular mechanisms governing betalain biosynthesis [14]. These discoveries have enabled the development of metabolic engineering strategies to enhance pigment production in both native and heterologous systems. Consequently, betalain biosynthetic genes have been successfully introduced into microorganisms, plants, and cultured cells, providing sustainable alternatives to conventional plant extraction and expanding the potential for industrial-scale pigment production [15].

Further developments in synthetic biology have contributed to advancing the research process by making it possible to create modular biosynthetic pathways and multigene expression systems. The creation of visual screening reporters like RUBY has not only helped in carrying out transformation screening but also proved the practicality of engineering betalains in plant biotechnology [16]. On the other hand, technologies like CRISPR/Cas have made it possible to precisely manipulate the biosynthetic pathways, transcription factors, and precursor biosynthesis networks responsible for the formation of betalains [17]. These technologies offer tremendous potential for optimizing metabolic flux, enhancing pigment accumulation, and improving stress tolerance in engineered organisms. Apart from whole-plant genetic engineering techniques, tissue culture systems have been identified as viable options for efficient pigment production in controlled conditions. Callus cultures, suspension cultures, and hairy root cultures offer scalable and environmentally controllable media [18] for the study of betalain biosynthesis and optimization of pigment production. They allow for strict control of the nutritional and phytohormonal requirements, as well as environmental stimuli affecting betalain accumulation. Additionally, tissue culture systems minimize the need for field cultivation and permit year-round production. Interventions based on nanotechnology have also been recognized as potential tools for promoting the production of secondary metabolites in plants. Nanoparticles can behave like abiotic elicitors that can induce oxidative stress responses and metabolic pathways involved in pigment formation. Several studies conducted using nanoparticles of selenium, zinc oxide, and titanium dioxide indicate that nanotechnology may play a pivotal role in pigment production due to signalling and stress-related metabolic responses in cells [19]. The impact of nanotechnology in the field of betalain biotechnology is in its nascent stage.

Despite substantial progress, several challenges continue to limit the efficient production of betalains. These include incomplete understanding of the regulatory networks governing betalain biosynthesis, limited precursor availability, stable transgene expression, large-scale production, and pigment stability. Addressing these challenges will require integrated multidisciplinary approaches that combine metabolic engineering, synthetic biology, omics technologies, and bioprocess optimization. As research in this field continues to expand, a comprehensive understanding of current strategies for enhancing betalain biosynthesis is essential for developing sustainable and commercially viable production systems.

Several comprehensive reviews have been published on the chemistry, occurrence, extraction, biological activity, and uses of betalains. Nevertheless, significant developments have taken place in areas of metabolic engineering, synthetic biology, multi-omics techniques, microbial bio-factories, and bioprocessing technology in the last few years. As opposed to previous reviews, the current review includes recent research in areas such as pathway engineering, elicitation, synthetic biology, tissue culture, nanotechnology, and omics-based identification of regulatory genes. Moreover, this review is a comparative assessment of plant-, plant cell-, yeast-, and bacterial-based production systems, industrial production, and commercial aspects, along with future prospects for sustainable betalain bio-factories.

## 2. Betalains: Discovery, Classification, and Biological Significance

Betalains are a distinct group of nitrogenous and water-soluble pigments that have gained much attention owing to their bright colours, bioactivities, and applicability as eco-friendly natural colourants. In contrast to many other plant pigments, betalains are found exclusively in the Caryophyllales, replacing anthocyanins from a functional perspective. Betalains are the cause of the distinctive red-violet and yellow-orange colours exhibited by many flowers, fruits, roots, stems, and leaves of plants like *B. vulgaris*, *Amaranthus* species, *Opuntia ficus-indica* (prickly pear), and *Hylocereus polyrhizus* (dragon fruit). The combination of their specific distribution pattern, distinctive chemical structure, and biological properties makes them one of the most intriguing groups of plant secondary metabolites [20]. Research into the structure and functionality of betalains can be traced back centuries, when beet pigments were isolated and tested for their dyeing properties. Initial studies on beet pigments were limited to beetroot due to its vivid colouration properties and long-standing role in dyeing applications, especially in food products. In addition, the name of beet pigments, namely “betalains,” was coined based on the name of the plant species that belongs to the Genus Beta, better known as red beets, which have remained primary sources for these compounds. The main red-violet pigment found in beetroot, named betanin, gained widespread recognition for its commercial importance as a natural dye, E162, that is applied to beverages, confectionery, dairy products, ice creams, and food products [7].

Betalains chemically represent water-soluble alkaloids that derive their formation from L-tyrosine amino acids and are defined by the presence of betalamic acid as the basic chromophoric molecule. Depending on the structural features and colouring properties, all betalains can be broadly grouped into two categories, namely betacyanins and betaxanthins [20,21]. Among them, betacyanins are those responsible for red to violet colouring and include betanin, amaranthin, gomphrenin, and phyllocactin, with a maximum absorbance ranging from 535 to 540 nm. On the contrary, the group of betaxanthins is responsible for yellow to orange colouring and represents a complex of betalamic acid and amino acids or amines, with the maximum absorbance ranging from 470 to 480 nm (Table 2) [22]. Mutual exclusivity between anthocyanins and betalains is a significant characteristic of these pigments. The production of anthocyanins and betalains by plants does not occur at once, as they do not co-exist within any organism [23]. This issue has fascinated researchers in the field of plant biology for decades. Though the exact reason for this exclusivity is still under study, there has been extensive research on this topic, which supports the idea that betalains may have developed as an alternative to anthocyanin pigment within Caryophyllales plants [23].

The presence of betalains in nature is rather restricted relative to other pigments found in plants. Nonetheless, they occur in many economically useful plants. Of them, *B. vulgaris* continues to be the main commercial source of betalains due to their abundant presence in this plant and easy cultivation [37]. Dragon fruit (*Hylocereus polyrhizus* and *H. costaricensis*) represents another commercially important plant, owing to the presence of high amounts of betacyanins in its deeply red-coloured flesh [38]. Prickly pear (*Opuntia ficus-indica*) also exhibits the presence of considerable quantities of indicaxanthin and betanin-like pigments [39]. Other examples are plants such as *Amaranthus*, *Celosia*, *Bougainvillea*, and *Basella alba*, as well as some cacti. The recent scientific literature mentions betalain-like substances, even in fungi and bacteria [40,41]. Betalains exhibit various physiological and ecological functions in plants. One of the most noticeable functions of betalains is pigment formation, which plays a significant part in attracting pollinators and seed dispersers. Flower and fruit colouration enhance reproductive success through enhanced visual appeal. Apart from ecological function, betalains play a key role in stress response and tolerance to environmental stressors [42,43,44]. There are several pieces of evidence indicating that betalain biosynthesis is induced during abiotic stress occurrence, including salt stress [45], light stress [46], ultraviolet stress [47], and extreme temperatures [48]. Betalains serve as excellent antioxidants, which can neutralize reactive oxygen species (ROS), preventing damage to cell structures [43]. The antioxidant activity of betalains is directly linked to the phenolic group and conjugated double bonds found in the structure [8]. In addition to their antioxidant activity, betalains also play an important role in the defence strategy of plants. Betalains’ presence in plants in response to pathogenic attacks or stressful environmental factors indicates their involvement in signal transduction pathways associated with stress tolerance [49,50]. Some studies have also suggested that betalains might be involved in stabilizing cellular membranes, preventing lipid peroxidation, and protecting photosynthesis-related organelles from oxidative stress [6,51].

The increased scientific interest in betalains can be mainly attributed to the biological activities associated with their medicinal effects. In the past 20 years, studies have found betalains to be antioxidants, anti-inflammatory, anticancer, hepatoprotective, cardioprotective, antimicrobial, neuroprotective, and antidiabetic [6,52]. Betanin and indicaxanthin have been studied extensively because of their unique ability to scavenge free radicals, which help to prevent inflammatory damage and protect the body from various health disorders. A number of in vitro and in vivo experiments have proven the anticancer activity of betalains. Beetroot and prickly pear extracts high in betanin content have exhibited anti-proliferative effects on different cancer cell lines such as colon, breast, prostate, liver, lung, cervical, and leukemia cancers [53]. The mechanisms underlying the action of betalains include apoptosis induction through mitochondrial pathways, activation of caspase enzymes, regulation of p53 signalling, and inhibition of cell proliferation [53,54]. Recent research has highlighted the epigenetic activities of betalains, which include alteration of DNA methylation and gene expression involved in cancer suppression [55]. Additionally, betalains have been reported to reduce angiogenesis, suppress inflammation, and inhibit metastatic progression in experimental models [56]. Betalains have thus found many new applications in the field of pharmaceuticals and nutraceuticals. The consumption of beetroot juice and extracts rich in betalains is becoming popular in functional food and dietary supplements owing to their various health benefits. Several clinical trials have demonstrated the potential of betalain-based products in improving cardiovascular health through reduced blood pressure, increased endothelial function, and decreased oxidative stress indicators [57].

## 3. Betalain Biosynthesis Pathway

**Figure 2:**
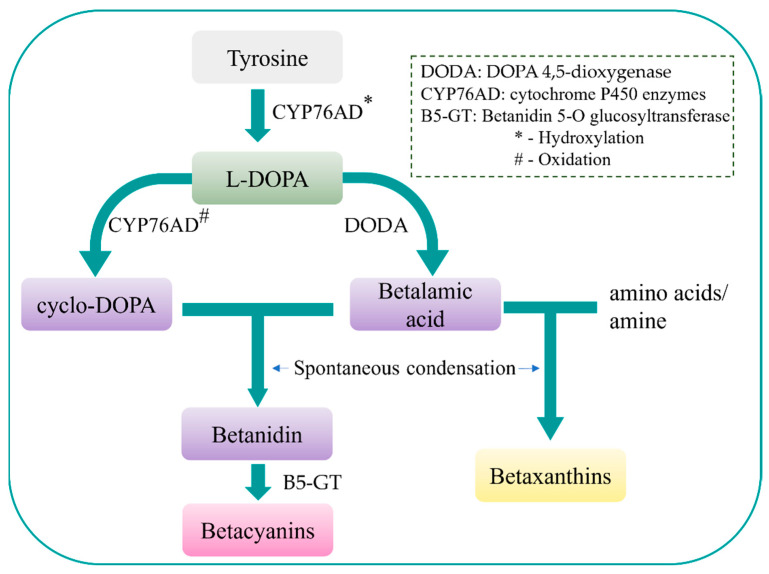
Simplified betalain biosynthetic pathway illustrating the conversion of Tyrosine to betalains through the sequential action of CYP76AD, DODA, and betanidin 5-O-glucosyltransferase (B5-GT).

Betalains have received much attention, owing to their distinct chemical makeup and functions in biology [58]. Betalains are synthesized from the aromatic amino acid L-tyrosine (Figure 2), formed in the shikimate pathway [22]. The first step entails the conversion of L-tyrosine to L-DOPA through hydroxylation via cytochrome P450 enzymes in the CYP76AD family that have tyrosine hydroxylase-like activity [59]. L-DOPA forms the intermediate compound in the two most critical steps for the synthesis of betalains [60]. In another pathway, the substrate L-DOPA undergoes oxidation into betalamic acid, the common chromophore of all betalains, via DOPA 4,5-dioxygenase (DODA) activity. Meanwhile, the alternative pathway involves the oxidation of L-DOPA into cyclo-DOPA via CYP76AD enzyme activity. The non-enzymatic or enzymatic condensation reaction between betalamic acid and cyclo-DOPA results in the production of betacyanins that provide red to violet colours. On the other hand, the condensation between betalamic acid and amino acids or amines produces betaxanthins that generate yellow to orange hues. Glycosylation is further utilized to modify the structure of betalains to increase solubility, stability, and accumulation of the pigment within vacuoles. Such structural modification is necessary for pigment deposition and performance [61]. The regulation of betalain biosynthesis depends on both genetic and environmental factors. Transcription factors belonging to the MYB, bHLH, and WRKY families have been observed to control the gene expression of biosynthetic genes in a tissue-specific and development-dependent manner [62]. The effect of environmental factors, such as light, temperature, and stress, on pathway regulation is exerted through alterations in gene expression and metabolic flow [63]. The betalain biosynthesis pathway involves relatively few enzymes compared with other plant pigment pathways. Biological synthesis of betalains depends, however, on strict control of metabolites, enzymes, and transcription factors. Research conducted recently has shown that the manipulation of enzymes, namely CYP76AD and DODA, and transcription factors can greatly increase the rate of betalain biosynthesis [64]. Overall, the betalain biosynthesis pathway appears to be an interesting example of a fairly simple but strictly controlled metabolic route. The detailed information about its enzymes and control factors will provide solid grounds for genetic modification of the pathway and enhancement of pigment yield, stability, and diversity.

## 4. Key Genes and Regulatory Networks Implicated in Betalain Biosynthesis

The process of betalain biosynthesis is controlled through a tightly controlled interplay between the structural genes responsible for the pathway enzymes involved in this process, as well as the regulatory genes responsible for regulating gene expression. Betalain biosynthesis requires only a few enzymes compared to any other pigment synthesis pathway in plants, but efficient synthesis requires regulation of these enzymes and their involvement in primary metabolism. The list of genes identified through various studies is summarized in Table 3.

### 4.1. Structural Genes in Betalain Biosynthesis

The core betalain pathway is primarily driven by three major classes of enzymes, namely, Cytochrome P450 enzymes (CYP76AD family), DOPA 4,5-dioxygenase (DODA), and Glucosyltransferases (GTs). The proteins belonging to the CYP76AD gene family have both a hydroxylation activity on L-tyrosine into L-DOPA and an oxidizing activity on L-DOPA into cyclo-DOPA, which is necessary to synthesize betacyanins [59,65]. Some specific functions of different forms, such as CYP76AD1, CYP76AD5, or CYP76AD6, can modify the proportion of synthesis between betacyanin and betaxanthin [59,66]. It is crucial for L-DOPA to degrade into betalamic acid to create the betalains. Betalamic acid acts as the chromophore that gives the pigment colour. It has been proven that DODA plays a significant role in producing both betacyanin and betaxanthins [67]. The enzymes responsible for the glycosylation of betalain precursors are the enzymes referred to as cyclo-DOPA 5-O-glucosyltransferase (cDOPA5GT) and betanidin 5-O-glucosyltransferase (B5GT). Glycosylation results in increased stability, solubility, and accumulation of the pigments in the vacuole [61]. Aside from these enzymes, which are directly responsible, additional genes that affect precursor availability, especially those involved in tyrosine synthesis and primary metabolism, play an indirect yet significant role in controlling betalain biosynthesis.

### 4.2. Regulatory Genes and Transcriptional Control

Expression of biosynthesis genes for betalains is strictly controlled by transcription factors that control gene activation during development and in response to environmental conditions. Of all transcription factors controlling such processes, MYBs are the best-studied. MYB transcription factors (e.g., BvMYB1, HuMYB132) are proteins that are important activators of genes related to the biosynthesis of betalains, as they attach themselves to promoters and increase transcription rates. Scientific studies have demonstrated the ability of MYB factors to directly activate genes like CYP76AD and DODA [68]. It has been shown that WRKY proteins are key players in the regulation between stress signalling and betalain production. For instance, certain types of WRKY proteins induce the expression of CYP76AD, which is responsible for the increased level of betalains when plants undergo fruit ripening and stress conditions [62]. Compared to MYB proteins, bHLH proteins are thought to work together with MYB regulators in regulating gene expression [69].

**Table 3:** Key genes in betalain biosynthesis.

Genes	Plant Species	Gene Functions	Reference
CYP76AD1	*Beta vulgaris*	Tyrosine to L-DOPA	[59]
DODA	*Amanita muscaria*	DOPA to betalamic acid	[42]
CYP76AD6	*Beta vulgaris* and *Mirabilis jalapa*	Betacyanin hydroxylation	[70]
CYP76AD5	*Beta vulgaris*	Betaxanthin hydroxylation	[59]
Cyclo-DOPA 5-O-glucosyltransferase	*Hylocereus undatus*	Cyclo-DOPA glycosylation	[66]
Betanidin 5-O-glucosyltransferase	*Beta vulgaris*	Betaxanthin glucosylation	[71]
CYP450s	*Beta vulgaris*	Betalain hydroxylation/methylation	[59]
AhMYB2	*Amaranthus hypochondriacus*	Betalain biosynthesis regulation	[72]
PuCYP76AD1	*Portulaca umbraticola*	Tyrosine to L-DOPA	[73]
cDOPA5GT	*Mirabilis jalapa*	Cyclo-DOPA to betanidin	[74]
5GT	*Dorotheanthus bellidiformis*	Betanidin glucosylation	[75]
Ugt	*Camellia japonica*	Betalain glycosylation	[76]
TYR	*Portulaca grandiflora*	Tyrosine to L-DOPA	[77]
DODA		L-DOPA to betalamic acid	[77]
CYP76AD	*Hylocereus undatus*	L-DOPA to cyclo-DOPA	[68]
GT	*Hylocereus polyrhizus*	Betanidin to betanin	[78]
HuMYB132	*Hylocereus undatus*	Betalain gene activation	[68]
DODA	*Hylocereus costaricensis*	DOPA to betalamic acid	[79]
CYP76AD1	*Amaranthus tricolor*	Tyrosine to cyclo-DOPA	[80]
DODA	*Amaranthus tricolor*	L-DOPA to betalamic acid	[80]
GT	*Amaranthus tricolor*	Betacyanin glucosylation	[80]
MYB1	*Hylocereus undatus*	Betalain biosynthesis regulation	[81]

### 4.3. Integration with Metabolic and Environmental Regulation

Betalain biosynthesis is related to primary metabolism, mainly L-tyrosine synthesis. The activities of enzymes involved in tyrosine synthesis, including arogenate dehydrogenase (ADH), affect precursor levels, thus affecting total pigment synthesis [82]. Differences in feedback inhibition of ADH activity have been shown to be correlated with an increase in betalain biosynthesis in specific plants [60,82]. Environmental conditions like light, temperature, and biotic/abiotic stress affect betalain biosynthesis [46]. Environmental conditions affect both the structural and regulatory gene expression in plants through stress-responsive transcription factors like WRKY [83].

### 4.4. Functional Significance and Engineering Implications

The simplicity of the architecture of the betalain biosynthesis pathway and the clear identification of its core genes render it highly susceptible to genetic and metabolic engineering approaches [84]. Research has shown that the concurrent expression of core structural genes, such as those coding for CYP76AD, DODA, and GTs, is adequate to reestablish the betalain synthesis process within non-native plants [85]. However, attaining high-level and constant betalain biosynthesis necessitates the coordinated control of all these factors. In general, interactions between biosynthetic proteins, transcription factors, and metabolic networks play a crucial role in determining the efficacy of the betalain biosynthesis process.

### 4.5. Functional Validation

The functional confirmation of genes involved in betalain biosynthesis was crucial in the validation of their respective functions in the pathway. The integration of different strategies, including the analysis of gene expression, RNA interference, Virus-Induced Gene Silencing (VIGS), gene overexpression experiments, and enzymology, has led to the authentication of roles of certain genes in the formation of pigments. Regarding the structural genes, CYP76AD genes have been confirmed to play an important role in the betalain biosynthesis pathway. It was shown that inhibition of CYP76AD gene expression resulted in reduced levels of betacyanin through the VIGS experiment conducted in *B. vulgaris* [70,86]. The enzyme (DODA) was also confirmed to play a crucial role in the pathway for synthesizing betalains. Knockdown experiments targeting the gene responsible for DODA activity in beet demonstrated an obvious absence of betalain pigments, implying that DODA is critical in the production of betalamic acid [87]. The research findings confirm the key role of the enzyme in the pathway by ensuring the production of the core chromophore needed to synthesize betacyanins and betaxanthins. Glucosyltransferases that catalyze the final stages of betalain production have also been studied using functional analysis. For instance, silencing B5GT genes and glucosyltransferases in *Hylocereus* species caused a significant decrease in betalain pigments, suggesting that glycosylation is important not only for the stability of betalain pigments but also for their effective accumulation in plants [88]. Aside from structural genes, transcription factors have been experimentally validated as important factors in controlling the biosynthesis of betalains. For example, overexpression and silencing of MYB transcription factors in *Amaranthus tricolor* have demonstrated their function in controlling the biosynthesis of CYP76AD and DODA [80]. This proves the importance of transcriptional regulation in controlling the activity and functioning of the pathway and thus obtaining pigments effectively. New advancements in understanding transcription factors have indicated that other factors, such as WRKY proteins, are also involved in the regulation of the expression of betalain biosynthesis genes. Function tests, including protein–protein interactions and promoter binding tests, have identified that these transcription factors induce the expression of structural genes, thus connecting environmental factors to the production of pigments. The above-mentioned experiments indicate that the production of betalains involves a tightly controlled network of enzymes and transcription factors involved. It is also shown that alteration in one particular factor affects the accumulation of pigments [80]. However, for enhanced betalain biosynthesis, a holistic system approach is required where several enzymes in the pathway are regulated together.

## 5. Current Status of Industrial Production and Challenges

The increasing global need for natural pigments has greatly boosted industrial interest in betalains as eco-friendly options to artificial dyes. Due to their brilliant colours, water solubility, antioxidant characteristics, and good safety record, betalains have become highly significant in the fields of food products, pharmaceuticals, nutraceuticals, cosmetics, and packaging. Currently, the industrial production of betalains heavily relies on the extraction of betalains from natural plants, where the main source is red beet (*B. vulgaris*) [89]. Some other recent sources of betalains are dragon fruit (*Hylocereus* spp.), prickly pear (*Opuntia ficus-indica*), amaranth, and cactus fruits [90].

Conventional extraction, purification, concentration, stabilization, and formulation are some of the major procedures used in the industrial synthesis of betalains. The use of conventional solvent extraction procedures that employ either water or aqueous ethanol as the extracting agent continues to be one of the widely utilized procedures owing to its ease, economy, and suitability for food applications [91]. However, several limitations exist with conventional extraction procedures, such as their low extraction yield, long extraction times, large quantities of solvent, and degradation of pigments. In order to eliminate the above-mentioned limitations, advanced extraction techniques have been introduced, such as ultrasonic-assisted extraction (UAE), microwave-assisted extraction (MAE), enzyme-assisted extraction (EAE), pressurized liquid extraction, and supercritical fluid extraction [92]. These technologies improve pigment recovery while minimizing thermal degradation and solvent usage. From all of the above methods, ultrasonic and microwave extraction processes have received much recognition, owing to their potential to break down cell walls, speed up the extraction process, and shorten extraction times. In addition, enzyme-assisted extraction can contribute to higher pigment extraction efficiency through the degradation of structural polysaccharides inside plants, leading to increased betalain extraction from cell interiors. Moreover, the development of green extraction techniques is also an emerging trend within recent industrial studies, aimed at reducing the environmental burden without compromising the pigment recovery rate [92,93]. These developments align with the growing demand for sustainable and eco-friendly industrial processing systems.

Even though there have been significant improvements in the extraction technologies of betalains, the use of these compounds in industrial applications is still greatly hindered by their poor physicochemical stability properties. In particular, betalains are prone to degradation from various environmental factors, such as temperature, illumination, oxygen, metal ions, hydration, and changes in pH levels [94,95]. Oxidation and hydrolysis reactions are frequent phenomena for betalains when exposed to certain environments, causing discolouration and reduced antioxidant effects. Moreover, food processing procedures, such as pasteurization and sterilization, frequently lead to severe destruction of betalains. Consequently, maintaining pigment stability throughout processing and storage represents one of the most critical challenges limiting broader industrial adoption. However, several solutions to stability concerns have been achieved through advances in encapsulation techniques. The use of biopolymers, proteins, liposomes, maltodextrin, gum arabic, as well as chitosan-based and nanocarrier encapsulation methods has proven highly effective in increasing the stability of betalains [96,97]. At the industrial scale, spray drying and freeze drying are the most commonly used techniques utilized in manufacturing betalain powders and extracts [96]. Encapsulation not only contributes to the increased stability of pigments but also enhances their bioavailability, controlled release, and suitability in different food matrices. Modern techniques of nanoencapsulation have enhanced betalains’ protection from oxidation and thermolysis processes.

One significant obstacle involved in industrial betalain production is the scarcity of abundant natural resources. While red beet is still a major commercially available resource, there are certain restrictions with using only one plant species as an industrial betalain source. Among others, such factors as seasonality, agricultural dependence, climate sensitivity, and variable pigment content may be listed. Moreover, the earthy taste of beetroot extract, mainly due to geosmin, can limit the use of betalains for food colouring purposes [61]. These limitations have stimulated research into alternative betalain-producing plants and engineered production systems capable of generating pigments with improved quality and stability.

## 6. Strategies to Enhance Betalain Production

Bioengineering approaches are gaining more attention in terms of sustainability in comparison with traditional agriculture for betalain extraction. In vitro tissue culture systems, such as callus culture, suspension culture, and hairy root culture, have shown promise in terms of controlled betalain production by using optimized and sterile conditions. Some of the benefits of using in vitro approaches include constant production throughout the year regardless of climatic fluctuations and regulation of metabolite synthesis.

### 6.1. Tissue Culture and Bioproduction

In vitro culture systems provide an effective and convenient system for betalain production and improvement. Various types of cultures, such as callus, cell suspension, and hairy root culture, have been extensively used to understand and optimize betalain biosynthesis in vitro. Callus culture is one of the most widely used methods for betalain biosynthesis. Studies conducted on Hylocereus costaricensis showed that betalain was highly accumulated in cell culture, with values up to 21.11 mg/g dry weight [98]. Likewise, callus cultures of *Bougainvillea* and *Amaranthus tricolor* have shown accumulation of betacyanin and betaxanthin pigments. Thus, undifferentiated cultures can be used for betalain production. Cell suspension culture is another effective method of betalain biosynthesis that can overcome the limitations associated with callus culture. A betalain content up to 23.27 mg/L was recorded in *Celosia argentea* [99]. These systems facilitate fine control of environmental factors like availability of nutrients, pH, and light to ensure optimization of pigment production. Hairy root cultures constitute another viable option for betalain production. Genetic stability, high rates of growth, and capability of producing secondary metabolites at the same level as entire plants make hairy root cultures a viable option for betalains. Hairy root cultures have been proven to be used successfully as reliable sources for betalains’ production in *B. vulgaris* [100]. Aside from the types of culture, other factors like elicitation, nutrient content, and environment also play vital roles in the regulation of betalain production. For instance, an increase in betalain content in callus cultures of *Hylocereus costaricensis* was achieved by means of amino acids and lighting [101]. Overall, tissue culture systems provide versatile and scalable platforms for betalain production (Table 4). These systems are particularly valuable for studying pathway regulation, screening genetic constructs, and developing commercial production strategies. Among the available in vitro systems, suspension cultures are more suitable for scale-up, whereas hairy root cultures provide superior genetic stability and long-term metabolite production.

### 6.2. Genetic Engineering Strategies

In recent years, genetic engineering has proven to be a very useful tool for increasing the production of betalains in both natural and heterologous plant hosts [108]. Various transformation methods have been used in order to enhance betalain production in plants through the manipulation of genes responsible for its synthesis, resulting in increased pigment formation, new pigment colouration, and betalain biosynthesis in new plant species [109]. *Agrobacterium*-based transformation continues to be the most popular choice for the functional analysis of betalain genes [110]. Early studies demonstrated the successful introduction of betalain biosynthetic genes into heterologous systems [71,111]. For example, the insertion of a betalain biosynthetic gene from Caryophyllaceae plants into tobacco will make it possible to study its role in the process of pigment formation. Similarly, *Chenopodium quinoa* transformation with CqCYP76AD1-1 and CqCYP76A1-2 genes showed that they are involved in the formation of pigments in hypocotyls [112]. Significant advances have been achieved by introducing several genes associated with betalain formation. For example, in carrots (*Daucus carota*), plasmids containing pYB:mCD (AomelOS, BvCYP76AD1S, BvDODA1S) and pYB:CDD (BvCYP76AD1S, BvDODA1S, and MjcDOPA5GTS) genes were introduced using the *Agrobacterium*-mediated method, resulting in the synthesis of betanin [110]. This shows that the simultaneous expression of all the genes in the pathway is needed for efficient pigment synthesis. Genetic engineering strategies can be used not only for structural genes but also for enhancing the level of pigments in many other plants. For example, betanin accumulation was increased by using the *Agrobacterium* strain GV3101:pMP90 in tomatoes [113]. Similarly, betalains were synthesized in anthocyanin-containing plants through the introduction of DOPA dioxygenase genes regulated by constitutive promoters [109]. Betalain production from hairy root culture systems, induced by infection of *Agrobacterium rhizogenes*, has also been investigated as an efficient system for producing betalains. It was reported that transformed hairy root cultures of *B. vulgaris* could produce betalain pigments similarly to those found in natural roots, thus serving as stable systems for betalain production [100]. Importantly, these pigments were accumulated only in root tissues and not in other plant organs, indicating that biosynthesis and accumulation mechanisms are controlled. Genetic transformation approaches have been further developed for use in ornamental plants. For example, in *Eustoma grandiflorum* (lisianthus), expression of CYP76AD1, DOPA dioxygenase (DOD), and cyclo-DOPA 5-O-glucosyltransferase (5GT) with a CaMV 35S promoter allowed the enhancement of betalain production in lisianthus flowers [114]. In general, these observations confirm the efficiency of multigene pathway construction rather than one-gene approaches for efficient and stable betalain production in plants. Thus, these results show that expression of betalain biosynthetic genes can be considered a promising approach for enhancing betalain pigment production in various species (Table 5).

### 6.3. Metabolic Flux Optimization and Synthetic Biology Design Principles for Enhanced Betalain Production

Engineering of the betalain biosynthetic process goes far beyond the overexpression of structural genes and includes the tuning of the entire metabolic pathway related to precursor synthesis, carbon metabolism, regulation, and metabolite trafficking inside cells. Although the genes coding for CYP76AD, DODA, and glucosyltransferases belong to the main machinery of betalain biosynthesis, there is ample evidence suggesting that the availability of precursors and metabolic flux control are the key factors in determining the production of pigments [84]. Being produced only from L-tyrosine, the availability of this aromatic amino acid often becomes the main limiting step for betalain biosynthesis.

The synthesis of tyrosine is controlled via feedback inhibition of arogenate dehydrogenase (ADH) and prephenate dehydrogenase (PDH) [122]. These enzymes are responsible for the regulation of amino acid balance in plants and microorganisms. The development of feedback-resistant forms of these enzymes represents one possible method for elevating tyrosine levels and re-channelling metabolism towards betalain biosynthesis [122]. Likewise, engineering of enzymes involved in the shikimic acid pathway, such as 3-deoxy-D-arabino-heptulosonate-7-phosphate synthase (DAHPS), chorismate mutase, and prephenate dehydrogenase, was suggested as a means for improving the availability of precursors [123]. Upstream approaches have proven to be effective in microorganisms and will become more popular in betalain bio-factories in the future.

Another key aspect is balancing the pathway. Overexpression of a single biosynthetic enzyme will not produce significant gains since the efficiency of the whole pathway depends on the coordinated efforts of various enzymes. Balanced expression of CYP76AD, DODA, and cyclo-DOPA 5-O-glucosyltransferase (cDOPA5GT) will avoid any metabolic bottleneck effects and will enable efficient production of betalains from intermediates. Modern synthetic biology techniques use multigene constructs, modular cloning technology, and gene stacking, as in the case of the RUBY reporter system, where various genes from the pathway can be placed under one transcription unit, resulting in visible betalain pigmentation in different plant species [115,124].

Promoter choice is yet another critical issue that affects the success of engineering efforts. Promoters like CaMV35S that provide constitutive expression ensure efficient transgene expression but may subject the plant to unnecessary metabolic stress [125]. On the other hand, promoters that ensure tissue-specific, developmental stage-specific, or induced expression of transgenes ensure targeted accumulation of betalains in economically valuable organs such as fruits, flowers, storage roots, or cell culture without affecting plant development [126,127]. Custom-made promoters with adjustable strength of expression have gained interest in regulating pathway expression and optimizing metabolism.

Subcellular localization also contributes to betalain production. Betalain biosynthesis takes place primarily in the cytoplasm, but pigments accumulate inside vacuoles where they are shielded from oxidative and enzymatic damage [128]. Transport mechanisms and compartment-specific expression of enzymes involved in the betalain pathway can prove to be interesting approaches.

Selection of the host for production is equally significant in assessing the economic feasibility of the process. Whole plants have natural precursor availability and pigment retention capability, but they need to be cultured for longer times and have regulatory limitations on genetically engineered crops. On the other hand, plant tissue cultures such as callus, suspension, and hairy root cultures allow controlled annual cultivation in sterile conditions and permit elicitor and bioreactor optimization, yet low productivity and the high cost of scale-up are problems. In turn, microbial hosts are fast-growing organisms, have easily manipulable genetics, and are suitable for industrial fermentation. Foreign microbial hosts like *Saccharomyces cerevisiae*, *Yarrowia lipolytica*, and *Escherichia coli* serve as promising options due to their fast growth and amenability to genetic manipulations. For instance, genetically modified *Saccharomyces cerevisiae* has been used to produce betanin, amounting to 17 mg L^−1^ directly from glucose, thus demonstrating for the first time that complete microbial biosynthesis of this commercially valuable natural colourant can be achieved when using yeast fermentation technology [129]. A recombinant strain of *Yarrowia lipolytica* expressing HpBAHD3 was able to produce 1.95 ± 0.024 g/L of the acylated betalain phyllocactin during 60 h of fed-batch fermentation, which underlines the potential of yeast as an industrial production host for betalain production [33]. Similarly, the development of a genetically engineered co-culture system using Escherichia coli facilitated the de novo synthesis of individual betaxanthins using glucose to produce 287.69 mg L^−1^ of histidine-betaxanthin [130].

### 6.4. Omics-Based Advances for Enhancing Betalain Production

Recent developments in omics techniques have made tremendous progress in the identification of genes, regulatory networks, and metabolic pathways responsible for the biosynthesis of betalains, offering a wealth of information for metabolic engineering and synthetic biology. Transcriptome studies have played an important role in the discovery of new structural genes and regulators related to the betalain biosynthesis pathway. A comparative transcriptome and metabolome analysis in different betalain-producing organisms led to the discovery of several new uncharacterized cytochrome P450s and glucosyltransferases playing roles in the tyrosine hydroxylation and betalain modification processes; on the other hand, the findings indicated that anthocyanin biosynthetic genes exist but might have lost their function during evolution to develop betalains [131]. In a similar way, the RNA-seq study of red-fleshed pitaya resulted in the identification of 33 candidate transcripts related to betalain biosynthesis, including nine critical genes of tyrosinase, DODA, cytochrome P450, and glucosyltransferase families contributing significantly to betalain production, establishing the molecular basis of betalain biosynthesis in pitaya [78].

The combination of genomics with transcriptomics and metabolomics has further contributed to knowledge regarding the regulation of pigments. For instance, the combination of the genome sequence of *Suaeda salsa*, along with transcriptomic and metabolomic comparisons, helped in the identification of four betacyanins and revealed that an increase in the accumulation of celosianin II and amaranthin leads to the production of the red colour phenotype [132]. Furthermore, a series of genes and transcription factors involved in the regulation of the betacyanin synthesis pathway have also been discovered. On the other hand, partial genome sequencing of *Hylocereus undatus* indicated that the genes involved in the betacyanin biosynthesis pathway were clustered in a 12 Mb region [133].

In addition to the discovery of biosynthetic genes, omics approaches have been used in revealing the molecular evolution mechanisms behind the mutual exclusivity of betalains and anthocyanins. Comparative genomics and transcriptomics of 357 species belonging to the Caryophyllales order revealed that the repeated loss of the anthocyanin transporter gene TT19, the downregulation of the late flavonoid biosynthetic genes, and the degradation of the MYB–bHLH–WD40 (MBW) transcriptional regulatory module played a key role in the replacement of anthocyanins with betalains during the course of evolution [23].

Taken together, integrated omics techniques such as genomics, transcriptomics, metabolomics, and comparative evolutionary analysis are offering an unparalleled insight into the biosynthesis and regulation of betalains. In addition to the identification of novel biosynthetic genes, transcription factors, and metabolic bottlenecks, these data provide reasonable targets for CRISPR-based genome editing and metabolic engineering. Therefore, multi-omics platforms will definitely be at the forefront of the development of betalain-producing crops and microorganisms of the next generation.

### 6.5. Elicitation Strategies for Enhancing Betalain Biosynthesis

It is important to note that elicitation has proven to be among the most useful methods for increasing the yield of secondary metabolites in the in vitro cultivation of plants. For example, in betalain biotechnology, it is common practice to employ elicitors in order to promote the formation of betalains through stimulation of the stress-activated metabolic pathways and gene transcriptions responsible for the biosynthesis of pigments. It has been proven that both abiotic and biotic elicitors are highly efficient for betalain accumulation in cell suspensions, as well as hairy root and callus cultures [134]. These elicitation techniques offer an effective solution to enhance the production of pigments in a controlled environment, which is especially significant in addressing the shortcomings posed by low efficiency in natural plants.

One of the best-studied groups of inducers of betalain biosynthesis is abiotic elicitors, which include plant hormones, heavy metals, mineral ions, osmotic stress compounds, lighting, heat shock, temperature changes, and signalling compounds like methyl jasmonate (MeJA) and salicylic acid (SA) [135]. The main mechanism of abiotic elicitors is the creation of controlled oxidative stress, which triggers the defence system of the cell and induces secondary metabolite synthesis. For betalain-producing species, abiotic elicitors lead to an increase in gene expression levels for key genes CYP76AD and DODA, resulting in betacyanins and betaxanthins production [48].

Among abiotic elicitors, methyl jasmonate is one elicitor that has gained significant attention owing to its ability to play an important role in the regulation of secondary metabolism. MeJA was reported to increase the synthesis of betalains in several in vitro cultures of plants. Betacyanins and betaxanthins were found to be increased to a greater extent by addition of MeJA at low concentrations in cultures of Bougainvillea calluses [135]. Furthermore, in the case of *Portulaca* suspension cultures, treatment with MeJA caused a notable increase in betalain biosynthesis [136]. Recent studies also indicate that methyl jasmonate-mediated elicitation in two species of *Alternanthera* enhances betalain biosynthesis through the activation of stress-responsive signalling pathways and transcriptional regulation of the key biosynthetic genes involved in secondary metabolism [137].

In addition, plant growth regulators also contribute to the process of betalain synthesis elicitation. The use of cytokinins, including 6-benzylaminopurine (BAP), has been found to promote cell growth and pigmentation in various betalain-forming cells. For example, when used at the appropriate concentrations, 6-benzylaminopurine (BAP) greatly enhances betalain biosynthesis in the exponential growth stage of *Celosia argentea* cell-suspension cultures [138].

The effects of heavy metals and micronutrient elicitors on betalain biosynthesis have also been positively observed. Copper sulphate (CuSO_4_), cobalt ions, and other microelements trigger oxidative pathways that promote secondary metabolic processes [139]. Copper ions are crucial, since some enzymes responsible for oxidation reactions, such as tyrosinase-like enzymes, require copper to function. Research conducted using the *Celosia argentea* cultures has shown that regulated CuSO_4_ addition effectively boosts betalain formation [140].

Recently, much attention has been paid to the synergistic effects of various elicitors. In traditional optimization experiments, only a single elicitor was used; however, when different elicitors were used in combination, there was improvement in betalain production. In a recent experiment on *C. argentea* var. plumosa, response surface methodology (RSM) was used to optimize combinations of elicitors, including BAP, MeJA, and CuSO_4_, in cell-suspension cultures. In optimized combinations of 2.28 µM BAP, 49.97 µM MeJA, and 6.71 µM CuSO_4_, the production level of total betalains increased by 3.9 times when compared to non-optimized cultures, reaching the maximum level of betalain concentration at 139.99 mg/L. Moreover, optimized cultures showed enhanced antioxidative effects. Moreover, the optimized cultures showed increased antioxidant potential, showing that elicitation can improve not just the pigmentation but biological activity as well [141].

The use of biotic elicitors has also demonstrated positive results in betalain biosynthesis. Elicitors that are produced by microorganisms, fungal cell walls, yeast extracts, and pathogen-related substances are capable of inducing defensive responses in plants and triggering the synthesis of secondary metabolites [142]. An experiment conducted on *Celosia cristata* cell cultures showed that the use of elicitors obtained from the cells of *Fusarium oxysporum* significantly increased the amount of betalains produced [143]. Similarly, chitosan has also been found to be an effective biotic elicitor, which helps boost betalain biosynthesis through the induction of defence response mechanisms, oxidation-based signalling, and metabolism of secondary metabolites [140]. Biotic elicitors usually induce the activation of signalling pathways that include jasmonates, salicylates, reactive oxygen intermediates, and mitogen-activated protein kinases (MAPK) cascades, thus controlling the transcriptional induction of biosynthesis genes.

Pigment biosynthesis is also influenced by physical elicitors like quality of light and ultraviolet radiation [47,144]. LED-based treatments are known to increase betalain formation in callus cultures of *H. costaricensis*. The exposure of *H. costaricensis* callus cultures to red LEDs caused an increase of 3.8 times for yellow calli and 4.8 times for red calli when compared with cool-white LED treatment, highlighting the significant effect of light quality on betalain biosynthesis [101]. Osmotic stress resulting from sucrose and salt treatments can lead to changes in metabolic pathways, thereby inducing betalain biosynthesis. Stress responses are often characterized by increased antioxidant activity and transcription factors responsive to stress stimuli.

Elicitation techniques have multiple benefits in terms of betalain biosynthesis in industry. They are cost-effective, easily deployable, and very suitable for in vitro cultivation and bioreactors. Furthermore, elicitation can be regulated for optimal pigment production without any permanent changes to the genes involved. Thus, elicitors are especially valuable in production processes that require the production of natural pigments with high levels of antioxidants and other functionalities.

### 6.6. Nanotechnology Approaches

Nanotechnology is one of the new strategies adopted to boost betalain biosynthesis in plants through the use of nanoparticles as elicitors. It is worth noting that nanoparticles exert their effects on plant metabolism through the induction of stress responses in plants, hence the stimulation of production of secondary metabolites like betalains. In this regard, recent studies show that nanoparticles such as nano-selenium (nano-Se), zinc oxide (ZnO), and titanium dioxide (TiO_2_) are capable of boosting betalain production in plant tissues. For example, pitaya treated with nano-selenium showed increased production of betalain due to the activation of enzymes in the phenylpropanoid pathway [145]. It also shows that nanoparticles may influence metabolism indirectly through regulatory mechanisms upstream. Likewise, ZnO and TiO_2_ nanoparticles have also been shown to improve betalain biosynthesis in callus cultures of *B. vulgaris* due to oxidative stress that stimulates secondary metabolism [146]. Such a stress-mediated pathway is considered one of the main routes through which nanoparticles affect betalain biosynthesis enhancement. Nanoparticles also present some benefits related to controlled delivery, enhanced bioavailability, and target specificity [147]. Nanoparticles’ small size and high surface area provide them with an effective way to interact with plants’ cells to modulate biochemical pathways. However, the use of nanoparticles in plants demands careful consideration because they can pose health risks to organisms in cases of incorrect dosing and other factors. It is necessary to consider nanoparticle types, concentrations, sizes, and exposure periods to obtain the desired effect. In general, techniques associated with nanotechnology are at the cutting edge in terms of betalain investigations. Combining nanoparticle induction with metabolic and genetic engineering could lead to synergetic improvement in betalain biosynthesis.

## 7. Challenges and Future Perspectives

**Figure 3:**
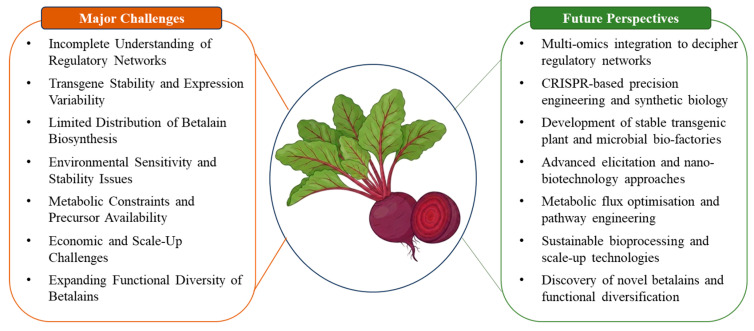
Challenges and future perspectives.

Though there have been many breakthroughs in the understanding and synthesis of betalains, some scientific and technical hurdles still impede their full utilization in agricultural biotechnology and industry (Figure 3). It is important to overcome these barriers for sustainable and economic betalain pigment production.

### 7.1. Incomplete Understanding of Regulatory Networks

One of the major hurdles in betalain research is the incomplete knowledge of the intricate regulatory networks that control the biosynthesis of these pigments. While there has been considerable success in identifying several structural genes and transcription factors such as MYB and WRKY, the overall network that includes upstream signals, gene-gene interactions, and feedback mechanisms still needs to be elucidated. Betalain biosynthesis is controlled by a number of endogenous and exogenous factors, both of which include developmental and environmental signals [61]. Nevertheless, how these factors coordinate and interact to regulate transcriptional activity remains unknown. Incomplete knowledge of the regulatory network poses a serious challenge to designing precise genetic modifications to enhance pigment synthesis. Future studies should emphasize using multi-omics approaches to unravel the regulatory network involved in betalain biosynthesis. Such system-level insights will enable the identification of key regulatory hubs and facilitate more efficient pathway engineering.

### 7.2. Transgene Stability and Expression Variability

One of the drawbacks of genetic engineering techniques is the instability of the expression of the introduced gene. There have been reports of variation in the accumulation of pigments in transgenic plants, owing to gene silencing, positional effects, and post-transcriptional gene regulation [148]. Instability of the introduced genes is a critical factor that limits the commercial viability of transgenic plant strains. Moreover, methods like the RUBY reporter, although extremely beneficial, are prone to yield false negatives in cases where the accumulation of pigment does not reach a detectable level [115,149]. This highlights the need for improved sensitivity and consistency in reporter systems. Future research should aim to create an expression system with stability, genomic safe harbours, and improved promoter and gene-stacking techniques. These improvements will enhance the reliability of genetic modifications and ensure consistent betalain production.

### 7.3. Limited Distribution of Betalain Biosynthesis

Betalains only occur naturally in the plant species belonging to the order Caryophyllales, and hence their production is naturally restricted in distribution. The expansion of betalain biosynthesis to new species poses a problem in that the genes responsible need to be effectively incorporated and expressed in the new organism. Gene expression experiments have proven that betalain biosynthesis can occur in foreign species, but efficient production and expression pose some problems due to host metabolism and the precursors involved [113]. Future research efforts would involve identification of the minimum gene sets needed to engineer pathways, as well as metabolic engineering in heterologous organisms and bio-factories. These approaches will enable broader application of betalains across diverse crop species.

### 7.4. Environmental Sensitivity and Stability Issues

Pigments are quite sensitive to different environmental parameters, including light, temperature, pH level, and enzyme-induced breakdown, affecting their stability during processing, storage, and use. This is a significant drawback that prevents using betalains in commercial applications, especially within the food and cosmetic industries [61]. Maintaining pigment stability and retaining colour intensity at the same time becomes an important problem. Different environmental variations may cause pigment breakdown, leading to a decrease in its functionality and esthetic appeal [150]. Future studies should pay attention to the creation of pigment stabilizers, implementation of encapsulation technologies, and modification of betalains. These strategies will improve the shelf life and applicability of betalain-based products.

### 7.5. Metabolic Constraints and Precursor Availability

The effective synthesis of betalains is influenced by the availability of precursors like L-tyrosine [151]. Nevertheless, metabolic limitations in primary metabolism can hamper the availability of precursors and hence hinder the synthesis process. Competition with other metabolic processes may also reduce the efficiency of betalain biosynthesis. Future studies need to focus on optimizing the metabolic flow in betalain biosynthesis and engineering pathways responsible for precursor synthesis. Integrating primary and secondary metabolism will be essential for achieving high-level betalain production.

### 7.6. Economic and Scale-Up Challenges

If betalains are to be economically feasible, there is a requirement for production platforms that can be scaled up to produce betalains in large quantities. However, existing production systems, such as plant extraction and in vitro culture systems, have limitations associated with economic feasibility, scalability, and reliability [138]. The major challenges ahead are those associated with coming up with economical production platforms, especially for commercial purposes. Future trends will focus on refining betalain production by using bioreactors, breeding high-betalain-producing plants, and combining agricultural and biotechnological approaches. These advancements will help bridge the gap between laboratory research and industrial application.

### 7.7. Expanding Functional Diversity of Betalains

Although betalains offer great potential applications, the diversity of their natural occurrence is still limited. Increasing the diversity of the structure and function of betalains is key to expanding their industrial and commercial use [64]. Future studies should aim at identifying new biosynthesis genes for betalains, designing new varieties of betalains, and integrating enzymes across different species. Such efforts will enable the development of customized pigments with improved properties and expanded applications.

For future research on betalains, the focus should be on integrating biotechnology advances with interdisciplinary methodologies. A correct combination of genetic manipulation, synthetic biology, nanotechnology, and omics studies can facilitate better control over betalain biosynthesis. Important trends in the future can include the creation of betalain factories in both plant and microbial hosts, the implementation of CRISPR technology for precision modification of metabolic pathways, and the incorporation of nanomaterials in elicitation to improve betalain biosynthesis, along with their utilization for improving stress resistance and crop quality. Through further development, betalains may be used as an essential component in sustainable pigment manufacturing, providing ecological substitutes for artificial pigments that not only improve human well-being but also drive innovation in industry.

## 8. Conclusions

Betalains are unique natural pigments that combine vibrant colouration with antioxidant, antimicrobial, and health-promoting properties, making them attractive candidates for applications in the food, pharmaceutical, cosmetic, and nutraceutical industries. Recent advances in metabolic engineering, synthetic biology, tissue culture, nanotechnology, and omics-based approaches have significantly expanded opportunities to enhance betalain biosynthesis and establish sustainable production platforms. Nevertheless, challenges related to pathway regulation, precursor availability, transgene stability, large-scale production, and pigment stability continue to limit their commercial exploitation. Future research should focus on integrating multi-omics, genome engineering, synthetic biology, and bioprocess optimization to develop efficient plant- and microbial-based bio-factories. Such multidisciplinary strategies will facilitate the replacement of synthetic colourants with safe, sustainable natural alternatives while supporting the development of functional foods, pharmaceuticals, and other high-value bioproducts.

## Figures and Tables

**Table 1:** Representative synthetic food colourants that can potentially be substituted by betalains and their reported toxicological effects.

Synthetic Dye	E Number	Colour	Potential Betalain Substitute	Reported Concerns
Allura Red AC	E129	Red	Betacyanins (Betanin)	Intestinal inflammation, DNA damage [9]
Carmoisine	E122	Red	Betacyanins	Loss of appetite, tachycardia, and drowsiness [10]
Erythrosine	E127	Red	Betacyanins	Oxidative stress [11]
Tartrazine	E102	Yellow	Betaxanthins	Neurodevelopmental toxicity, oxidative stress [12]
Sunset Yellow FCF	E110	Yellow	Betaxanthins	Genotoxicity reports [13]

**Table 2:** Chemical structures, descriptions, and absorption characteristics of key intermediates and representative betalain pigments.

S.No.	Compound Name	Description	Chemical Structure ^#^	Absorption Spectra—λmax * (nm)
1.	Tyrosine	A non-essential aromatic amino acid that plants (specifically within the order Caryophyllales) convert into betalains [8]	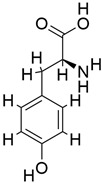	-
2.	L-3,4-dihydroxyphenylalanine (L-DOPA)	A central amino acid precursor that branches directly into the production of all betalain pigments [8]	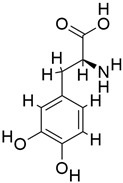	-
3.	cyclo-DOPA	A critical amino acid derivative that acts as the core building block for red-violet plant pigments called betacyanins [8]	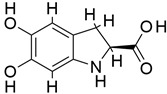	-
4.	Betalamic acid	Betalamic acid is the fundamental structural core and chromophore for all betalains [8]	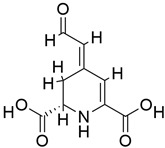	424 [24]
5.	Betanidin	A glycone betacyanin is produced through the condensation of betalamic acid with cyclo-DOPA [25,26]	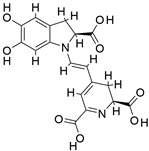	541 [26]
6.	Betalains	Nitrogen-containing water-soluble plant pigments derived from betalamic acid. Betalains are divided into Betacyanins (red to violet pigments) and Betaxanthins (yellow to orange pigments) [8]	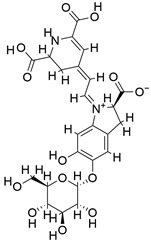 General structure	-
7.	Betacyanin	The red to violet subclass of betalains is synthesized from betalamic acid and cyclo-DOPA derivatives [8,27]	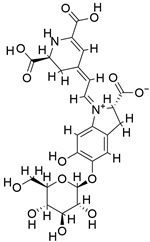	538 [24]
8.	Betaxanthin	Yellow to orange betalain pigments are synthesized from betalamic acid and various amino acids or amines [8,27]	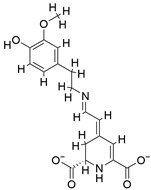	480 [24]
9.	Betacyanins/Betanin	The primary red-violet betacyanin pigment found in beetroot is glucosylated betanidin [28]	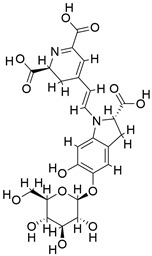	530–532 nm [28]
10.	Betacyanin/Amaranthin	Betacyanin pigment consisting of betanidin with a glucuronosyl-glucose disaccharide moiety [25]	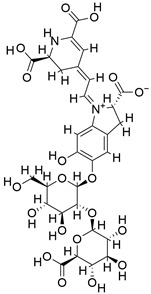	535 [29]
11.	Betacyanins/Isobetanin	The C15 epimer of betanin occurs naturally alongside betanin in plants that are rich in betalains [25]	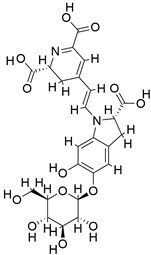	536 [30]
12.	Betacyanins/Hylocerenin	An acylated betacyanin derivative, present in pitaya (dragon fruit) fruits [31]	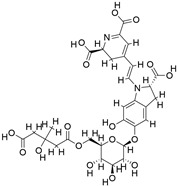	535 [32]
13.	Betacyanins/Phyllocactin	The malonylated derivative of betanin serves as a major red pigment in dragon fruit [33]	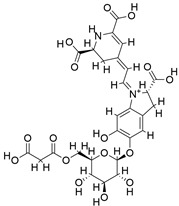	535 [32]
14.	Betacyanins/Gomphrenin	The betacyanin pigment group is characterized by glucosylated betanidin derivatives, which are commonly found in Gomphrena species [34]	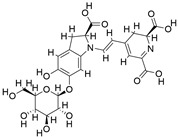	543 [35]
15.	Betaxanthin/Indicaxanthin	Yellow betaxanthin is produced through the condensation of betalamic acid with proline [8]	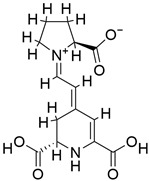	482 [36]

* λmax: wavelength of maximum absorbance. Absorption maxima (λmax) may vary slightly depending on solvent, pH, temperature, and experimental conditions. ^#^ Chemical structures were obtained from the PubChem database (National Center for Biotechnology Information, NCBI) and are presented for illustrative purposes.

**Table 4:** Tissue culture-based betalain production.

Plant Species	Tissue Culture Technique Used	Results	Reference
*Hylocereus costaricensis*	Cell suspension cultures	21.11 mg/g DW betalain	[98]
*Bougainvillea cv. Bhabha*	Callus culture	0.57 mg/g FW and 0.45 mg/g FW betacyanin and betaxanthins	[102]
*Hylocereus costaricensis*	Callus culture	18.13 mg/g DW of betalain	[101]
*Chenopodium quinoa*	Callus culture	9.55 µg/g FW betacyanin and 0.98 µg/g FW betaxanthin	[103]
*Stenocereus queretaroensis*	Cell cultures	82.77 and 78.76 µg/g FW of betacyanin and betaxanthin in sqR1 line	[104]
*Gomphrena globosa*	Callus culture	0.371 ± 0.035 mg/mg FW of betacyanin	[105]
*Amaranthus tricolor*	Callus culture	4.04 mg/100 g FW of betacyanin	[106]
*Pereskia aculeata*	Cell suspension cultures	4.10 ± 0.47 mg/100 mL of betalains	[107]
*Pereskia aculeata*	Callus culture	1.14 ± 0.18 mg/100 mL of betalains	[107]

**Table 5:** Genetic engineering studies using betalain pathway.

Plant	Construct	Method	Outcome	Reference
*Nicotiana benthamiana*	pCBL101-RUBY	*Agrobacterium* mediated	Purple-pigmented areas were visible	[16]
*Maize inbred B104*	pCBL101-RUBY	*Agrobacterium* mediated	Dark purple, light-purple and purple-striped leaves were visible	[16]
*Plukenetia volubilis*	DR5::RUBY	*A. rhizogenes* strain K599	Low rate of RUBY transgenic hairy roots were reddish	[115]
*Gossypium hirsutum*	35 S-RUBY	CaMv constitutive promoter	Pink cotton fibre	[116]
*Arabidopsis*	35S:RUBY	*Agrobacterium*-mediated floral dipping	Transformed plants displayed patches of red colour	[85]
*Daucus carota*	p35S:RUBY	Callus transformation	Red-violet callus formation	[117]
*Daucus carota*	p35S:RUBY-S	Callus transformation	Yellow-orange, red, and red-violet callus formation	[117]
*Nicotiana tabacum*	RUBY		The RUBY-transformed shoots displayed a reddish colour.	[118]
*Nicotiana benthamiana*	35S:RUBY	*Agrobacterium tumefaciens*	Intense red pigmentation	[119]
*Arachis hypogaea*	pBinBarRuby	*Agrobacterium tumefaciens*-mediated transformation	Transformed callus turned red-violet, and transgenic shoots also exhibited a red-purple colour	[120]
*Lotus corniculatus*	RUBY	*Agrobacterium rhizogenes*	Red hairy roots were produced	[121]

## Data Availability

No new data were created or analyzed in this study.

## Abbreviations

The following abbreviations are used in this manuscript: CYP, Cytochrome P450; DOPA, 3,4-Dihydroxyphenylalanine; DODA, DOPA 4,5-dioxygenase; GT, Glycosyltransferase; DFR, Dihydroflavonol 4-Reductasec; Ugt, UDP-dependent Glycosyltransferase; TYR, Tyrosinase; 1MYB, Myeloblastosis Transcription Factor; RUBY, betalain reporter construct; bHLH, Basic Helix-Loop-Helix Transcription Factor; WRKY, WRKY Domain-containing Transcription Factor; ADH, arogenate dehydrogenase; PDH, prephenate dehydrogenase; CRISPR-Cas9, Clustered Regularly Interspaced Short Palindromic Repeats—CRISPR Associated Protein 9; CaMV, Cauliflower Mosaic Virus.
